# HSP27 Alleviates Cardiac Aging in Mice via a Mechanism Involving Antioxidation and Mitophagy Activation

**DOI:** 10.1155/2016/2586706

**Published:** 2016-03-23

**Authors:** Shenglan Lin, Yana Wang, Xiaojin Zhang, Qiuyue Kong, Chuanfu Li, Yuehua Li, Zhengnian Ding, Li Liu

**Affiliations:** ^1^Department of Geriatrics, First Affiliated Hospital with Nanjing Medical University, Nanjing 210029, China; ^2^Department of Anesthesiology, First Affiliated Hospital with Nanjing Medical University, Nanjing 210029, China; ^3^Department of Surgery, East Tennessee State University, Johnson City, TN 37614, USA; ^4^Department of Pathophysiology, Nanjing Medical University, Nanjing 210029, China

## Abstract

Aging-induced cardiac dysfunction is a prominent feature of cardiac aging. Heat shock protein 27 (HSP27) protects cardiac function against ischemia or chemical challenge. We hypothesized that HSP27 attenuates cardiac aging. Transgenic (Tg) mice with cardiac-specific expression of the* HSP27* gene and wild-type (WT) littermates were employed in the experiments. Echocardiography revealed a significant decline in the cardiac function of old WT mice compared with young WT mice. In striking contrast, the aging-induced impairment of cardiac function was attenuated in old Tg mice compared with old WT mice. Levels of cardiac aging markers were lower in old Tg mouse hearts than in old WT mouse hearts. Less interstitial fibrosis and lower contents of reactive oxygen species and ubiquitin-conjugated proteins were detected in old Tg hearts than in old WT hearts. Furthermore, old Tg hearts demonstrated lower accumulation of LC3-II and p62 than old WT hearts. Levels of Atg13, Vps34, and Rab7 were also higher in old Tg hearts than in old WT hearts. Additionally, old Tg hearts had higher levels of PINK1 and Parkin than old WT hearts, suggesting that mitophagy was activated in old Tg hearts. Taken together, HSP27 alleviated cardiac aging and this action involved antioxidation and mitophagy activation.

## 1. Introduction

Intrinsic cardiac aging is defined as slowly progressive functional declines and structural changes with age, in the absence of major cardiovascular risks such as hypertension, diabetes, hypercholesterolemia, and smoking [1, 2]. However, intrinsic cardiac aging can increase the vulnerability of the heart to both endogenous and exogenous stressors, ultimately increasing cardiovascular mortality and morbidity in elderly individuals [1, 2]. Therefore, interventions to combat cardiac aging not only will improve the healthspan of the elderly, but also can extend their lifespan by delaying cardiovascular disease-related deaths [3].

The phenotypes of the aging heart have been well characterized; however, the molecular mechanisms underlying cardiac aging are not fully understood [3]. Studies indicate that the pathogenesis of cardiac aging involves multiple molecular mechanisms, including oxidative stress, impaired autophagy, metabolic changes, dysregulated calcium homeostasis, and activation of neurohormonal signaling [3, 4]. Indeed, the reactive oxygen species (ROS) content significantly increases in the aged heart, while mitochondrial overexpression of catalase (an important antioxidative enzyme) improves the aging-induced decline in cardiac function and prolongs the lifespan of mice [5, 6]. Intracellular ROS in the aged heart are mainly generated from damaged mitochondria [1]. In normal conditions, damaged mitochondria are selectively degraded through autophagy, or mitophagy, a term coined by Lemasters [1, 7]. Unfortunately, autophagy is progressively impaired over time [8].

Heat shock protein 27 (HSP27) is an ubiquitously expressed member of the small heat shock protein subfamily with a molecular weight of 27 kDa in humans and 25 kDa in rodents. Studies demonstrated the involvement of HSP27 in various biological functions, including the responses to oxidative stress, heat shock, and hypoxic/ischemia injury [9, 10]. Of particular interest to this study, we and others showed that overexpression of HSP27 protects cardiac function against cardiac injuries induced by ischemia/reperfusion, myocardial infarction, inflammation, and doxorubicin [9, 11–13]. The mechanisms that contribute to cardioprotection by HSP27 involve the antioxidative capacity, suppression of inflammatory responses, improvement of cardiomyocyte survival, and activation of autophagy and mitochondrial activity [9, 11–17]. It is possible, therefore, that overexpression of HSP27 protects the heart from aging-induced injury.

In this study, we examined the effects of HSP27 on cardiac aging using transgenic (Tg) mice with cardiac-specific expression of HSP27. We observed an improvement in cardiac function and decreases in the levels of cardiac aging markers in old Tg mice compared with age-matched wild-type (WT) controls. This action of HSP27 involves the antioxidative capacity and activation of mitochondrial autophagy (mitophagy). Our results suggest that management of HSP27 expression may serve as an alternative intervention to alleviate cardiac aging.

## 2. Materials and Methods

### 2.1. Antibodies and Reagents

A primary antibody against HSP27 was obtained from Stressgen (Victoria, British Columbia, Canada). Antibodies against p16, p53, Atg13, Nix, phosphor-p53 (Ser15), p21, and GAPDH were obtained from Bioworld Technology Inc. (Louis Park, MN). Antibodies against LC3, p62, Rab7, and Vps34 were from Cell Signaling Technology (Beverly, MA). Antibodies against Parkin, PINK1, and BNIP3 were from Abcam (Cambridge, MA). An antibody against ubiquitin was from Santa Cruz Biotechnology (Dallas, TX). An antibody against dinitrophenyl was from Sigma (Saint Louis, MO). A BCA protein assay kit was obtained from Pierce (Rockford, IL). 2′,7′-Dichlorofluorescein (DCFH) was obtained from Merck (Darmstadt, Germany).

### 2.2. Animals

Tg mice with cardiac-specific expression of the human* HSP27* gene (HSP27 Tg) driven by the *α*-MHC promoter were developed using mice with a CBA/BL6 genetic background and back-crossed with C57BL/6 mice for more than ten generations as described previously [9, 13]. In the experiments, 24-month-old HSP27 Tg mice and gender-matched WT littermates served as old mice. In echocardiographic measurements, 2-month-old HSP27 Tg mice and gender-matched WT littermates served as young controls. Mice were bred and maintained at the Model Animal Research Center of Nanjing University and maintained in the Animal Laboratory Resource Facility at Nanjing University. All the experiments were performed in compliance with the Guide for the Care and Use of Laboratory Animals published by the US National Institutes of Health (NIH Publication, 8th Edition, 2011). The animal care and experimental protocols were approved by the Nanjing University Committee on Animal Care. All experiments were performed in compliance with the international guidelines on the ethical use of animals.

### 2.3. Echocardiography

Two-dimensional echocardiographic measurements were performed using the Vevo 770 system equipped with a 35 MHz transducer (Visualsonics, Toronto, Canada) as in our previous methods [18]. Mice were anaesthetized by inhalation of 1.5–2% isoflurane. The measurements were performed by an observer blinded to the treatment. The parameters were obtained in M-mode tracings at the papillary muscle level or pulse doppler evaluation of the bicuspid valve and averaged using at least five continuous cardiac cycles.

### 2.4. Masson's Trichrome Staining

Heart tissues at the papillary muscle level were collected for paraffin sectioning. Masson trichrome staining was used to analyze fibrosis according to previously described methods [18]. The staining was observed using a microscope at a magnification of 400x. The percentage areas of fibrosis in the ventricular myocardium were measured using a software program (Olympus, Japan).

### 2.5. DCFH Assay

ROS levels were measured using the fluorescent indicator DCFH, as previously described [9, 19]. When DCFH is added to a tissue homogenate, ROS in the homogenate will lead to the oxidation of DCFH, producing the fluorescent product DCF. In our experiments, 1 *μ*M DCFH was incubated in a volume of 1 mL containing 10 *μ*g of cardiac cytosolic proteins, and fluorescence was recorded following incubation for 1 h using a fluorometer (Synergy HT, BIO-TEK) at an excitation wavelength of 485 nm and an emission wavelength of 535 nm.

### 2.6. Protein Carbonylation

Ventricular tissues were homogenized in lysis buffer (0.3 M sucrose, 0.03 M nicotinamide, and 0.02 M EDTA, pH 7.4) and centrifuged at 10,000 ×g for 5 min at 4°C. A volume of 500 *μ*L containing 100 *μ*g of cytosolic proteins was incubated with 2 mM DNPH in the dark for 1 h. The DNPH-treated proteins were used to detect protein carbonylation by Western blotting.

### 2.7. Immunofluorescence Staining

Hearts were harvested, fixed in 4% paraformaldehyde overnight, and processed for paraffin-embedded sectioning. The sections were then dewaxed, rehydrated, subjected to antigen retrieval, and blocked. Subsequently, the sections were incubated with the appropriate primary antibodies at 4°C overnight, followed by FITC- or Cy3-conjugated secondary antibodies for 60 min at room temperature. *α*-actinin was used to indicate cardiomyocytes. Hoechst 33342 was used to counterstain nuclei. The staining was observed and images were captured using a fluorescence microscope at a magnification of 400x. In LC3-II staining experiments, LC3-II granules were counted and expressed as the number of LC3-II dots per mm^2^ of myocardial tissues.

### 2.8. Western Blotting

Hearts were collected from 24-month-old mice. Western blotting of these samples was performed as described in our previous studies [9, 18]. Briefly, cellular proteins were prepared from ventricular samples, separated by 10% SDS-PAGE, and transferred onto Immobilon-P membranes (Millipore). The membranes were probed with appropriate primary antibodies, followed by incubation with peroxidase-conjugated secondary antibodies. The signals were detected by enhanced Pierce chemiluminescence. The blots for GAPDH served as a loading control. The signals were quantified by scanning densitometry, and the results from each experimental group were expressed as relative integrated intensity compared with that of controls.

### 2.9. Statistical Analysis

Data are presented as mean ± standard deviation. Groups were compared using Student's two-tailed unpaired *t*-test or one-way analysis of variance followed by a post hoc procedure (Tukey's test) for multiple range, as appropriate, with SPSS 13.0 software (SPSS Inc., Chicago, IL). Statistical significance was set at *P* < 0.05.

## 3. Results

### 3.1. HSP27 Alleviates the Aging-Induced Decline in Cardiac Function

**Figure 1:**
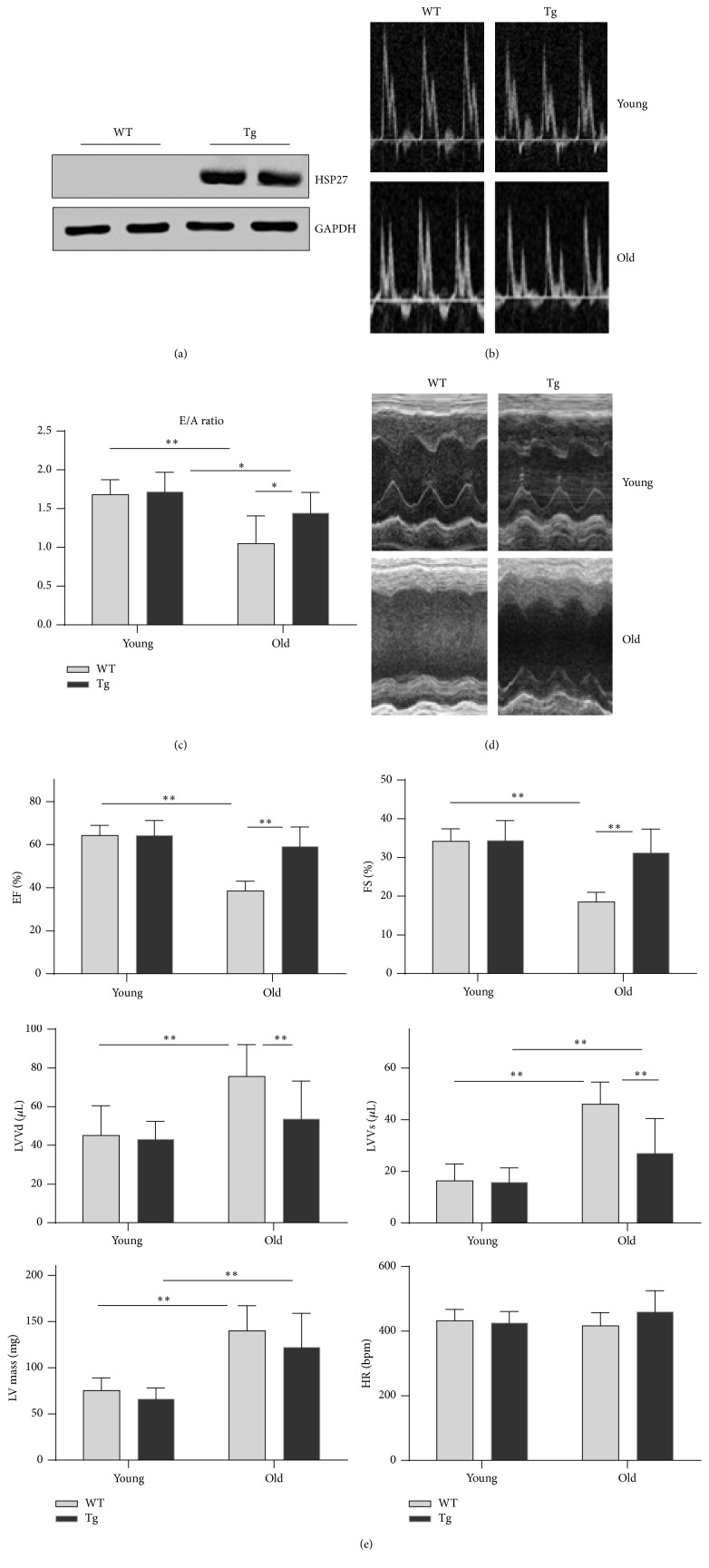
HSP27 alleviates the aging-induced decline of cardiac function. (a) Expression of HSP27 in the hearts of HSP27 Tg mice. The hearts were collected from adult WT and Tg mice. Protein extracts were prepared for immunoblot analysis against HSP27. The blots against GAPDH served as loading controls. Note that the primary antibody against HSP27 had no cross-reaction with murine homologue of HSP27. *n* = 3 per group. (b) Representative images of pulse doppler of the bicuspid valve echocardiography. *n* = 6, 9, 3, and 14 in young WT, young Tg, old WT, and old Tg groups, respectively. (c) E/A ratios. ^*∗*^ *P* < 0.05 and ^*∗∗*^ *P* < 0.01. *n* = 6, 9, 3, and 14 in young WT, young Tg, old WT, and old Tg groups, respectively. (d) Representative images of M-mode tracing of echocardiography. *n* = 10, 10, 7, and 38 in young WT, young Tg, old WT, and old Tg groups, respectively. (e) EF, FS, LVVd, and LVVs values. ^*∗∗*^ *P* < 0.01. *n* = 10, 10, 7, and 38 in young WT, young Tg, old WT, and old Tg groups, respectively.

To evaluate the roles of HSP27 in cardiac aging, we generated Tg mice expressing the human* HSP27* gene. Successful expression of the transgene was confirmed by Western blot analysis, which demonstrated abundant expression of HSP27 in Tg mouse hearts, but not in WT controls (the antibody against HSP27 did not cross-react with murine HSP25) (Figure 1(a)).

The decline in cardiac function is a hallmark of cardiac aging [20]. Therefore, we examined cardiac performance using two-dimensional echocardiography in old (24-month-old) and young (2-month-old) mice. Cardiac diastolic function was reflected by the ratio of peak early to late diastolic filling velocity (E/A ratio). Old WT mice demonstrated a significant decrease in the E/A ratio by 37.5% compared with young WT mice (*P* < 0.01) (Figures 1(b) and 1(c)). Furthermore, the E/A ratio was decreased by 15.9% in old Tg mice compared with young Tg controls (*P* < 0.05). However, the aging-induced decrease in the E/A ratio was significantly attenuated by 37.4% in Tg mice compared with WT mice (*P* < 0.05).

Cardiac systolic function was reflected by ejection fraction (EF), fraction shortening (FS), and left ventricular (LV) volumes at end-diastolic and end-systolic phases (LVVd and LVVs, resp.). Old WT mice demonstrated significant decreases in EF (40.0%) and FS (45.9%) and increases in LVVd (67.4%) and LVVs (181.0%) compared with young WT mice (*P* < 0.01) (Figures 1(d) and 1(e)). By contrast, EF, FS, and LVVd remained normal in old Tg mice compared with young Tg controls. Compared with old WT mice, EF and FS were significantly increased and LVVd and LVVs were significantly decreased in old Tg mice (*P* < 0.01).

The LV mass was significantly higher in both old WT mice (85.7%) and old Tg mice (84.7%) than in their genotype-matched young controls (*P* < 0.01). However, the LV mass did not differ between old WT and old Tg mice. The heart rate did not differ between the young and old groups (Figure 1(e)).

### 3.2. HSP27 Decreases the Levels of Aging Markers (p53 and p16) in the Myocardium of Old Mice

**Figure 2:**
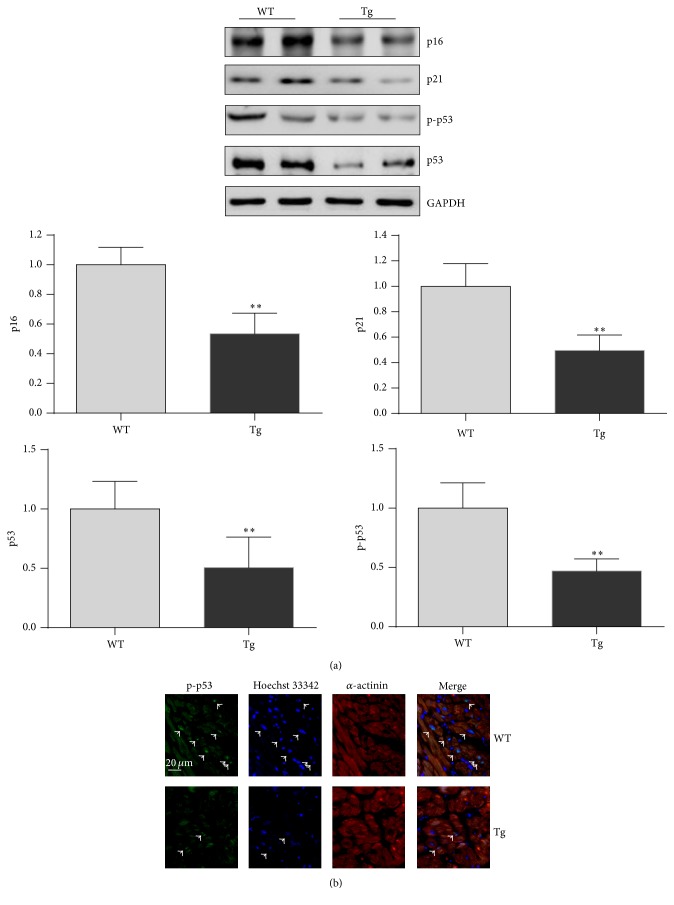
HSP27 decreases the expression of p16 and p53 in hearts of old mice. The hearts were collected from 24-month-old mice. Protein extracts were prepared for immunoblot analysis against p16 and p53. The blots against GAPDH served as loading controls. ^*∗∗*^ *P* < 0.01 versus WT controls. *n* = 3 per group.

The cell cycle regulators p16 and p53 play important roles in cellular senescence and therefore serve as aging markers [21, 22]. Therefore, the expression levels of p16 and p53 were examined in hearts of old mice. The results of immunoblot analysis are shown in Figure 2(a). Levels of p16 and p53 were significantly reduced by 46.5% and 49.4%, respectively, in hearts of old Tg mice compared with hearts of old WT mice (*P* < 0.01).

Expression of p21 is regulated by p53. Hearts of old Tg mice demonstrated a significantly lower level of p21 than hearts of old WT mice (*P* < 0.01) (Figure 2(a)).

To determine the activation of p53, we examined the phosphorylation level of p53 at Ser15 (p-p53). The p-p53 level was significantly lower in hearts of old Tg mice (53.0%) than in hearts of old WT mice (*P* < 0.01) (Figure 2(a)). To determine whether the activation of p53 occurred in cardiomyocytes, we performed fluorescence immunohistochemistry of cardiac tissues. Positive staining of p-p53 was localized in the nuclei of cardiomyocytes in the hearts of both old WT and old Tg mice (Figure 2(b)).

### 3.3. HSP27 Attenuates Fibrosis in the Interstitial Myocardium of Old Mice

**Figure 3:**
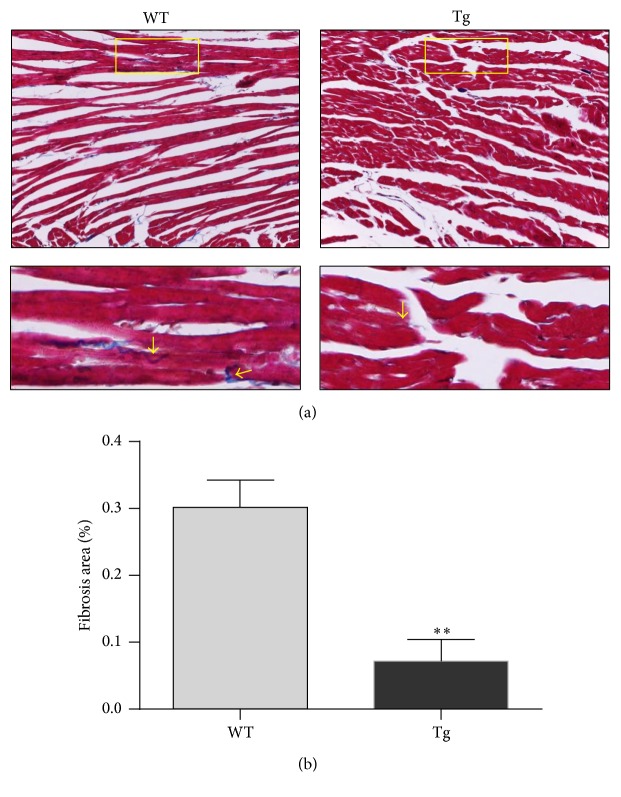
HSP27 reduces fibrosis deposition in interstitial myocardium of old mice. The hearts collected from 24-month-old mice were prepared for paraffin-embedded sectioning. Masson's trichrome staining was performed on the sections to evaluate the fibrosis (indicated by arrows). Higher magnification images of the boxed areas are shown in the down panels. Representative images from three independent experiments are shown (a). The quantification of fibrosis was shown (b). ^*∗∗*^ *P* < 0.01 versus WT controls. *n* = 3 per group.

Compromised diastolic function is associated with myocardial interstitial fibrosis [23]. We examined myocardial fibrosis in old mice using Masson's trichrome staining. Obvious fibrosis was observed in the myocardial interstitium of old WT mice (indicated by arrows) (Figures 3(a) and 3(b)). By contrast, there was significantly less fibrosis in old Tg mouse hearts than in old WT mouse hearts. Fibrosis was significantly reduced by 76.2% in old Tg mouse hearts compared with old WT mouse hearts (*P* < 0.01).

### 3.4. HSP27 Decreases the ROS Content in the Myocardium of Old Mice

**Figure 4:**
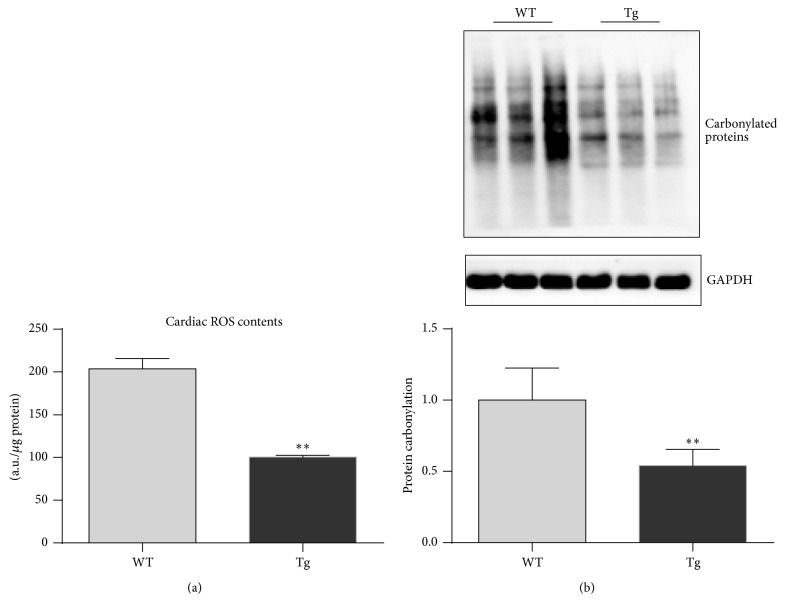
HSP27 decreases ROS contents in the hearts of old mice. The hearts were collected from 24-month-old mice. DCFH assay was performed to evaluate intracellular ROS contents. The relative contents of ROS were expressed as the arbitrary units per *μ*g protein. ^*∗∗*^ *P* < 0.01 versus WT controls. *n* = 3 per group.

ROS are one of the critical players in the development of cardiac aging [1]. To determine whether ROS are involved in the protective effect of HSP27 against the aging-induced decline in cardiac function, we measured ROS contents in the myocardium of old mice. The ROS content was 2038.6 and 752.4 units in old WT and old Tg mouse hearts, respectively (Figure 4(a)). Therefore, hearts of old Tg mice demonstrated a significantly lower ROS content (by 63.1%) than old WT mouse hearts (*P* < 0.01).

Protein carbonylation was also examined in hearts of old mice. Protein carbonylation was significantly lower in hearts of old Tg mice (46.1%) than in hearts of WT mice (*P* < 0.01) (Figure 4(b)).

### 3.5. HSP27 Decreases the Accumulation of LC3-II and p62 in Old Hearts

**Figure 5:**
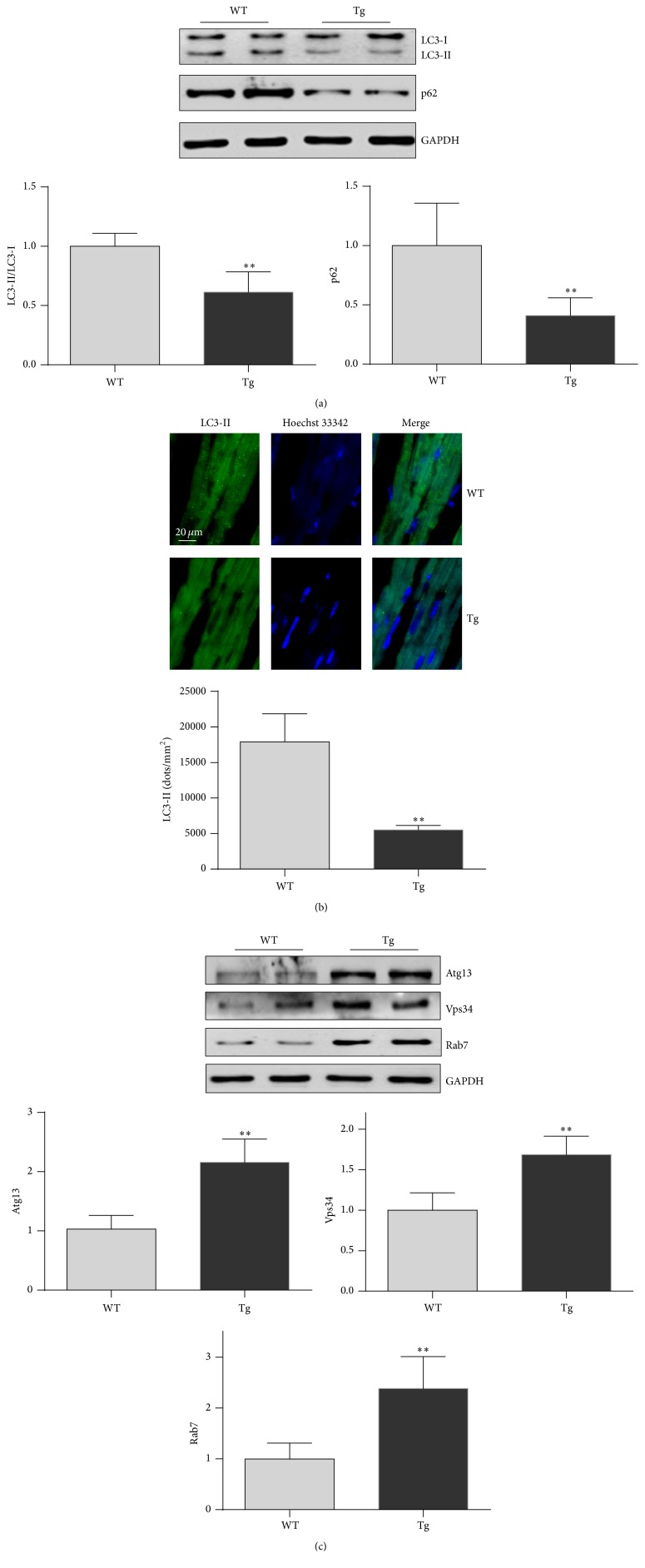
HSP27 increases autophagic markers in the hearts of old mice. The hearts were collected from 24-month-old mice. Protein extracts were prepared for immunoblot analysis against LC3 and p62 (a) and against Atg 13, Vps34, and Rab7 (b). The blots against GAPDH served as loading controls. ^*∗∗*^ *P* < 0.01 versus WT controls. *n* = 3 per group.

Activation of mitochondrial autophagy (mitophagy) alleviates cardiac aging [24]. We first examined LC3 conversion and p62 protein degradation, two widely used markers of autophagic flux [25], in hearts by immunoblot analysis. The level of LC3-II was significantly decreased by 38.7% in old Tg mouse hearts compared with old WT mouse hearts (*P* < 0.01) (Figure 5(a)). Immunostaining of LC3-II confirmed the observation made in immunoblot analysis (Figure 5(b)). By contrast, the p62 protein level was reduced by 59.0% in old Tg mouse hearts compared with old WT mouse hearts (*P* < 0.01). These data suggest that autophagic flux was significantly higher in old Tg mouse hearts than in old WT mouse hearts.

Atg13, Vps34, and Rab7 play important roles in the formation and maturation of autophagosomes [26–28]. Hearts of old Tg mice demonstrated significant increases in the levels of Atg13 (112.7%), Vps34 (68.5%), and Rab7 (138.4%) compared with hearts of old WT mice (*P* < 0.01) (Figure 5(c)).

### 3.6. HSP27 Increases PINK1 and Parkin Expression in Old Hearts

**Figure 6:**
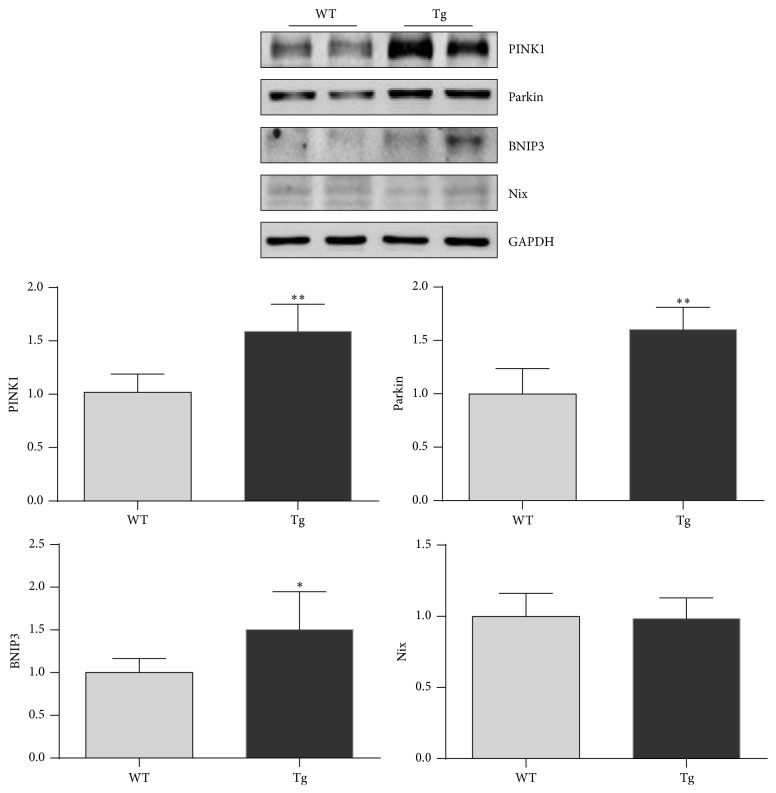
HSP27 increases PINK1 and Parkin expression in the hearts of old mice. The hearts were collected from 24-month-old mice. Protein extracts were prepared for immunoblot analysis against PINK1 and Parkin. The blots against GAPDH served as loading controls. ^*∗*^ *P* < 0.05 and ^*∗∗*^ *P* < 0.01 versus WT controls. *n* = 3 per group.

PINK1 and Parkin are important for mitophagy induction [29]. The levels of PINK1 and Parkin were increased by 57.1% and 60.5%, respectively, in hearts of old Tg mice compared with hearts of old WT mice (*P* < 0.01) (Figure 6). The mitophagy receptors BNIP3 and Nix were also examined by immunoblot analysis. The results demonstrated a significantly higher level of BNIP3 in hearts of old Tg mice compared with hearts of old WT mice (*P* < 0.05). The Nix levels were comparable between the two genotypes.

### 3.7. HSP27 Reduces the Content of Ubiquitin-Conjugated Proteins

**Figure 7:**
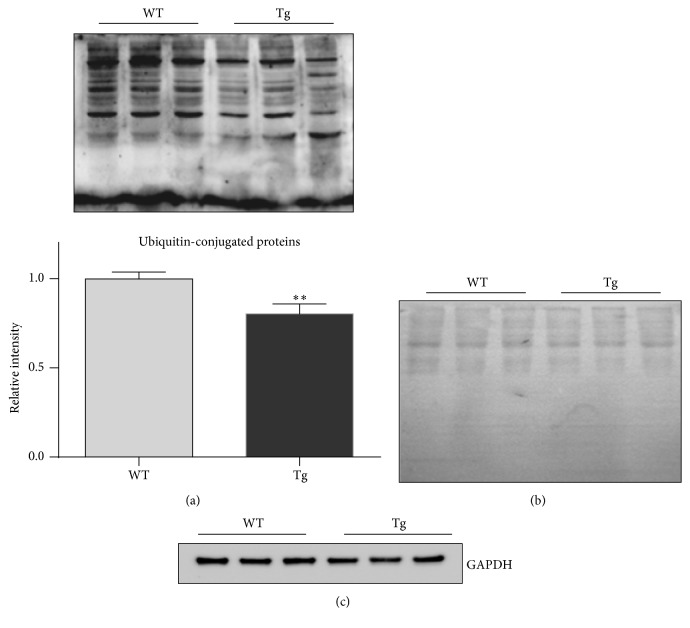
HSP27 decreases the levels of ubiquitin-conjugated protein in the hearts of old mice. The hearts were collected from 24-month-old mice. Protein extracts were prepared for immunoblot analysis against ubiquitin (a). The Coomassie blue-stained gel served as loading control (b). ^*∗∗*^ *P* < 0.01 versus WT controls. *n* = 3 per group.

Autophagy is an important approach for the degradation of ubiquitin-conjugated proteins and damaged organelles including mitochondria [30]. We examined the levels of ubiquitin-conjugated proteins. Hearts of old Tg mice exhibited significantly lower levels of protein ubiquitination (76.2%) than hearts of old WT mice (*P* < 0.01) (Figure 7(a)). As protein loading controls, the SDS-PAGE gel was stained with Coomassie blue (Figure 7(b)) and the blot was probed for GAPDH (Figure 7(b)).

## 4. Discussion

The significant finding of this study is that cardiac-specific expression of HSP27 attenuated the aging-induced decline in cardiac function of mice. Reduced expression of cardiac aging markers, less interstitial fibrosis, and lower levels of ROS and ubiquitin-conjugated proteins were observed in old Tg mouse hearts compared with old WT mouse hearts. Moreover, old Tg mouse hearts showed significantly decreased accumulation of LC3-II and p62 proteins and increased expression of Atg13, Vps34, Rab7, PINK1, and Parkin compared with old WT mouse hearts. Taken together, these results suggest that HSP27 alleviates cardiac aging and that this action involves antioxidation and mitophagy activation.

Cardiac aging is associated with compromised cardiac function. In humans, the LV early diastolic filling rate progressively slows after the age of 20 years, and therefore by 80 years of age the rate is reduced, on average, by up to 50% [20]. EF, a parameter indicative of LV systolic function, can decrease by more than 20% in healthy old individuals [20]. Consistent with this, aged animals also demonstrate an impairment in cardiac function [1, 31]. In this study, cardiac systolic and diastolic functions were reduced and LV diameters were increased in 24-month-old WT mice. However, the aging-induced decline in cardiac function and the enlargement of LV diameter were attenuated in HSP27 Tg mice. These results suggest that HSP27 overexpression improves the aging-induced impairment of cardiac performance.

While senescence is reflected by phenotypic changes in functions and structures, the molecular mechanism underlying cellular senescence involves p16- and p53-mediated pathways [32]. p16 and p53, two important tumor suppressors, mediate senescence through inhibiting the action of cyclin-dependent kinases, which in turn leads to G1 arrest of the cell cycle [32]. Indeed, an inverse relationship between the expression levels of p16/p53 and senescence was well demonstrated by studies of both humans and animals [32, 33]. Therefore, p16 and p53 are indicated to be signatures of in vivo senescence [33, 34]. In this study, significantly lower levels of p16 and p53 expression were detected in old Tg mouse hearts compared with old WT mouse hearts, suggesting that aging-induced decline of cardiac function was alleviated by HSP27 overexpression.

The well-known free radical theory of aging, first proposed by Harman in 1956, posits that intracellular production of ROS is the major determinant of lifespan [1, 35]. Indeed, antioxidative interventions, such as overexpression of mitochondrial catalase, significantly improve cardiac systolic and diastolic functions and decrease protein oxidative damage in aged mice [36]. Moreover, antioxidation reduces the expression of p16 and p53 [37, 38]. In this study, old Tg mouse hearts exhibited significantly lower levels of ROS and protein carbonylation than old WT mouse hearts. Supporting this, we and others revealed that HSP27 possesses an antioxidative capacity in myocytes and other cells [13, 39]. Taken together, our results suggest that antioxidation was involved in the protective effect of HSP27 against aging-induced decline of cardiac function.

Autophagy is an evolutionarily conserved process involved in the degradation of damaged proteins, mitochondria, and other organelles [40]. The intracellular accumulation of damaged macromolecules and organelles is a feature common to all aged cells [41]. The postmitotic nature of cardiomyocytes makes these cells especially prone to the accumulation of macromolecular and organellar oxidative damage over the lifetime, which means that more autophagic activation is required to clean up this harmful cellular “garbage” in order to maintain cardiomyocyte homeostasis [1]. Unfortunately, autophagic activity in cardiomyocytes declines with age, while impaired autophagy has multiple consequences including decreased contractile function and increased arrhythmia risk [42, 43]. Evidence suggests that stimulation of autophagy via genetic deletion of endothelin receptor A rescues the aging-induced impairment of cardiac contractile dysfunction [43]. In this study, we observed increased activation of autophagy, which was reflected by decreased accumulation of LC3 and p62, in old HSP27 Tg mouse hearts compared with old WT mouse hearts. Collectively, these data suggest that autophagic activation is involved in the protective effect of HSP27 against cardiac aging.

Mitophagy refers to selective autophagy for mitochondrial degradation. Mitochondria are both the main source of ROS generation and targets of ROS attack. Due to the high reliance of cardiomyocytes on mitochondrial energy metabolism, the heart is exposed to a high burden of ROS throughout the lifetime [1]. Activation of PINK1 and Parkin plays a critical role in turnover of damaged mitochondria through mitophagy. PINK1 kinase activity and its mitochondrial localization are prerequisites for inducing translocation of the E3 ligase Parkin to depolarized mitochondria. Subsequently, Parkin mediates the formation of two distinct polyubiquitin chains to recruit autophagy receptors [44]. Thus, PINK1 signals mitochondrial dysfunction to Parkin, and Parkin promotes the elimination of damaged mitochondria. Moreover, p53 inhibits Parkin-mediated mitophagy [45]. In this study, we observed significant increases in PINK1 and Parkin levels in old Tg mouse hearts compared with old WT mouse hearts. By contrast, a lower level of p53 was detected in old Tg mouse hearts compared with old WT mouse hearts. Taken together, our data suggest that activation of mitophagy contributes to the alleviation of cardiac aging by HSP27.

In summary, our data provide functional links between HSP27 expression and improvement of the aging-induced decline in cardiac function, which suggests that management of HSP27 expression serves as a potential intervention for alleviation of cardiac aging.
